# The relationship between moral distress in nurses and ethical climate in selected hospitals of the Iranian social security organization

**DOI:** 10.18502/jmehm.v12i8.1339

**Published:** 2019-08-04

**Authors:** Mina Bayat, Mohsen Shahriari, Mahrokh Keshvari

**Affiliations:** 1 *MSc* * Student, * *School of Nursing and Midwifery,* *Isfahan University of Medical Sciences, Isfahan, Iran.*; 2 *Associate Professor, Nursing* * & Midwifery Care Research Centre,* *School of Nursing and Midwifery, Isfahan University of Medical Sciences, Isfahan, Iran.*; 3 *Assistant Professor, Nursing* * & Midwifery Care Research Centre,* *School of Nursing and Midwifery, Isfahan University of Medical Sciences, Isfahan, Iran.*

**Keywords:** Moral distress, Nurses, Ethical climate, Hospital

## Abstract

The present study was conducted to determine the relation between nurses’ moral distress and the ethical climate in selected hospitals of the Iranian Social Security Organization (ISSO). This descriptive-analytical correlational study was conducted in 6 hospitals under the coverage of the Iranian Social Security Organization in 2016. Three hundred nurses were selected by convenience sampling method. Data were gathered using Corley’s Standard Moral Distress and Olson’s Hospital Ethical Climate Scales. Data were analyzed using SPSS software version 19.

The mean score of the nurses’ moral distress was 1.94 ± 0.66, which is considered moderate. The mean score of ethical climate was 88.97, indicating desirable ethical climate in these hospitals. The frequency score of moral distress had a unilateral reverse correlation with the total score of ethical climate as well as its dimensions, including colleagues, patients, hospitals and physicians. The score of the intensity of nurses’ moral distress also had a unilateral reverse correlation with the total score of ethical climate and the scores of the hospital and physicians dimensions.

These results emphasized the importance of creating a positive ethical climate to decrease moral distress as well as the need for professional interventions to increase support in moral issues.

## Introduction

Generally, providers of health-related services encounter various moral issues and problems on a regular basis (1). Changes in the healthcare system lead to an increased need for ethics laws, policies and therapeutic instructions, and highlight the accountability of the healthcare personnel. Also, occupational pressures and raised levels of social expectations increase health-care providers’ level of moral distress (2). On the other hand, developments in technology and medicinal interventions in patient care, especially when the results are uncertain, would escalate moral conflicts (3). These moral problems could lead to stress in caregivers and consequently cause physical, emotional, mental and social moral distress (1).

As important members of the health team, nurses play an essential role in providing competent, responsive and moral care. However, nursing care is mostly provided in a context filled with moral conflicts and challenges (4). Moral distress is frequently discussed in relation to occupational satisfaction, occupational burnout and interactions in nursing relationships (5). In addition, various personal and structural factors could be effective in causing moral distress (6).

Moral distress is a disturbing mental imbalance caused by recognition of a morally correct action that cannot be performed due to organizational barriers such as lack of time, supervisors’ unwillingness, physicians’ inhibiting power structure, organizational policies and legal considerations (7).

Moral distress is a common phenomenon in nursing practice that can cause conflicts when encountering patients and providing quality care. On the one hand, moral distress may disrupt the process of achieving care system objectives and consequently have an adverse effect on the health pattern of the society (8); on the other, it can create mental and physical problems for nurses, which may influence their occupational satisfaction and their willingness to remain in the profession, and eventually the quality of care (9). Nurses have reported moral distress as a result of changes in human resources and the health system as well as increased social demands (2).

Moral distress affects not only nurses’ professional life by disturbing their focus and creating feelings of inefficiency, but also their personal life by causing mood disorders and irritability (5). Study results have shown that increased levels of moral distress could cause medical errors, harm, burnout, excessive fatigue and reluctance to help patients (10). Furthermore, extreme disappointment and occupational dissatisfaction might lead to collateral violence and in general, an insecure work environment (11).

In most studies, the emphasis has been on determining the relation between nurses’ personal characteristics and moral distress, and less attention has been paid to the organizational environment and inter-organizational relationships (12). Ethical climate indicates a common understanding of the organizational activities associated with moral decisions and inter-organizational issues such as power, trust and human relationships (13). Fry et al. showed that the lower the level of the hospital ethical climate is, the higher the severity of perceived moral distress and its complications will be (14). Also, Borhani et al. revealed a reverse relationship between perceived moral distress in nurses and their perception of the ethical climate (8). Hart showed that a negative ethical climate is related to nurses’ decision to leave their job or the nursing profession (15). Corley et al. reported that 25% of the studied nurses had left their positions due to moral distress. Aiken et al. also found that 40% of the nurses were not satisfied with their work environment and 1 out of 3 nurses aged under 30 planned on leaving their jobs within the next year (16). Also about half of the nurses (44%) reported a decline in the quality of the care they provided. It seems that in order to determine the causes of moral distress, occupational satisfaction and nurses’ willingness to change their place of duty, the ethical climate needs to be improved (17).

There is evidence about the higher occurrence of moral distress in certain occupational situations (1). Rice et al. reported that nurses working in oncology and organ transplant wards experience moral distress more than other nurses (18). Another study reported that health community nurses and nurses working in mental hospitals experience lower levels of moral distress (19). Anke et al. showed that in providing end-of-life care, due to lack of internal independence, sometimes nurses are not able to work based on their values, which might lead to moral distress (5). Also, there is evidence about the effect of colleagues’ support (4), supervision (20) and ethical climate on moral distress, all of which are influenced by the perceptions of the nursing staff, organizational viewpoints and management of moral issues (6).

Most of the studies in this field have been conducted on the quiddity, prevalence and personal determinants of moral distress. Few studies have been conducted on occupational factors involved in occurrence or non-occurrence of moral distress (21) and the effect of the workplace on the occurrence of moral distress (22). Therefore, it is necessary to evaluate these factors comprehensively and provide effective solutions to prevent moral distress among nurses (23). According to surveys, all related research in Iran has been conducted in hospitals affiliated with medical universities and the issue has not been investigated in hospitals under the coverage of the Iranian Social Security Organization (ISSO).

The Iranian Social Security Organization (ISSO) is a public insurance institution whose main mission is to cover wage and salary workers (compulsory) and self-employed individuals (optional). The population covered by this organization is about 12 million insured people and more than 2 million pensioners, and reaches 37 million including family members who receive health care.

By law, the ISSO is a public nongovernmental establishment that is not reliant on government resources, and the major part of its funding comes from the premium provided through participation of the insured and the employer. For this reason, its assets pertain to segments covered in successive generations and cannot be merged with any governmental or nongovernmental organization or institution. The core of this organization is the tripartite participation of employers, insured persons and the government in various fields of policy- and decision-making as well as financing.

Hospitals covered by the ISSO do not operate under the supervision of medical universities and therefore do not have a strong academic and educational background. Since a large portion of health services in Iran is provided by this organization and many nurses are hired there after graduation, research in this field, which has so far been outdated, seems to be necessary. Moreover, the results of such studies can contribute to a better understanding of this huge part of the community health system. Additionally, since the researcher is employed as a nurse in one of the ISSO hospitals, she is familiar with the study environment and relevant authorities, which has facilitated the research process and helped to better reflect the actual experiences of the participants. For these reasons, the present study was conducted to determine the relationship between moral distress in nurses and the ethical climate in selected hospitals of the ISSO in two cities.

## Method

The present study was a descriptive-analytical correlational study conducted in 6 hospitals under the coverage of the ISSO (4 in Tehran and 2 in Isfahan) during 2016. The sample size for this study was calculated to be 255 participants, and after considering a 20% sample loss, the number was increased to 300. The share of each hospital was determined based on the number of the nurses working there. At first the sampling method was quota, and then samples were selected using convenience sampling from the wards of the selected hospitals. The inclusion criteria were having at least a bachelor’s degree in nursing and having a minimum one-year work experience at the hospital.

We had to obtain permission from the ethics committee of the Isfahan University of Medical Sciences (No. 395230) and make arrangements with the treatment management of the ISSO in the study environment. After explaining the goals of the study and presenting the proposal, we received approval to enter the selected hospitals. To attract the participation of nurses, a meeting was held with the presence of hospital management and nursing managers to explain the process and methods of the study, and participants were assured that the results of the study would be presented to hospital authorities, optionally. After preparing a list of the nurses in each ward, the number of participants from each ward was determined based on the quota to each hospital, and the questionnaires were distributed among the nurses through convenience sampling.

To provide ethical considerations, the participants’ written consent was obtained at the beginning of the queries to make certain of their agreement to participate in the research. As the next step, the goals and nature of the study were explained to the participants and they were assured of confidentiality of their information and the voluntary nature of participation. Subsequently, questionnaires were distributed among the nurses in three working shifts based on the allocated quota for each ward. To answer the questions of the participants, the researcher remained at each ward during an entire working shift. The questionnaires were distributed among the participants at the beginning of each working shift when there was enough time to talk, and the manner of answering the questions was explained to them. The participants were asked to complete the questionnaire before their next working shift at the latest, and then place it in the envelope that was given to them and hand it to their head nurse. Due to the sensitivity of the issue and in order to ensure data collection precision, the researcher personally did the entire work for all the 300 participants, and was present in the research environment both in Tehran and Isfahan.

A three-part questionnaire was used for gathering the data. The first part was about demographic characteristics including age, gender, marital status, educational level and profession-related information such as type of employment, working ward and hospital, work experience and the number of overtime hours. The second part included Olson’s Ethical Climate Scale, designed and psychometrically evaluated by Olson in 1995 to measure hospital ethical climate. Olson determined the validity of the questionnaire using content validity index (CVI) at 87% and its reliability at 91% using Cronbach’s alpha (Olson 1995). This scale was translated and used in a study by Hariri et al. in 2011 (24), and its content validity index and reliability were measured using internal consistency and test-retest methods (content validity was 0.89). In the present study, the translated tool from Hariri’s study was used. This scale has 26 items scored from almost never (1) to almost always (5) using a 5-point scale. Thus the score of each questionnaire ranges between 26 and 130, with higher scores indicating more positive ethical climates. This questionnaire contains 5 factors that evaluate nurses’ perceptions about their colleagues (questions 1, 10, 18 and 23), patients (questions 2, 6, 11 and 19), managers (questions 3, 7, 12, 15, 20 and 24), hospital (questions 4, 8, 13, 16, 21 and 25), and physicians (questions 5, 9, 14, 17, 22 and 26).

The third part aimed to measure moral distress and was first designed by Corley in 1995. This scale has 24 items, each presenting a stressful situation and asking the respondents to score the moral distress they would experience in each case. Corley’s scale shows the frequency and severity of moral distress in nurses based on a 5-point Likert scale from 0 to 4. To determine the severity of distress, options varying from “it causes no distress for me” (score of 0) to “it causes great distress for me” (score of 4) were used. To determine the frequency of perceived distress, options varying from “I have never experienced moral distress” (score of 0) to “I have experienced a lot of moral distress” (score of 4) were used. The lowest score (0) indicated the minimum perceived distress at the intended situation and the highest score (96) indicated the maximum perceived distress at the intended situation. The total score of the questionnaire would be categorized as low (0 to 24), moderate (24.1 to 48), high (48.1 to 72) and very high (72.1 to 96). Also, at the end of the questionnaire, there was a three-choice question to evaluate nurses’ intention to leave the nursing practice. In this study, the translated tool from Borhani’s study was used. The validity of this scale was measured to be 100% by Borhani et al. (2014) (8), using content validity index (CVI). The reliability of the tool was approved using test-retest and Cronbach’s alpha. Data were analyzed using SPSS software version 19 and descriptive statistics including mean, percentage, standard deviation, tolerance and inferential statistics to determine the relation and correlation between qualitative and quantitative variables including Pearson’s correlation coefficient, Spearman’s correlation coefficient, and one-way variance analysis.

## Results

Three hundred questionnaires were completed by the nurses and then analyzed. According to the results, the mean age of the participants was 37 years, 73.3% were female, 78.7% were married, and 84% had a bachelor’s degree. In terms of employment, 58% had ordinary organizational positions, 76% were officially employed, and 88.3% did not work in other hospitals. Also, 86% had a work experience of 15 to 19 years and the mean of their overtime hours per month was 77.4, while 76% had to work extra obligatory hours. Most of the participants (56%) were working rotational shifts, 7.51% had passed ethics courses, and 61 percent were chosen based on their interest in nursing (Table 1).

According to the results, the mean score of perceived moral distress in nurses was 1.94 ± 0.66, which indicated moderate moral distress. Among the items related to the frequency of occurrence of moral distress, the item of “Giving nursing care to a patient under ventilation with no hope for living” had the highest (2.1 ± 9.34), and the item of “When patient’s death is inevitable, I speak to the family about organ donation” had the lowest frequency of moral distress (1 ± 1.16).

Among the items related to the severity of moral distress, the item of “Due to the large number of patients, I cannot provide high quality care to all of them” had the highest (with a mean of 2.1 ± 84.26), and the item of “I accept the physician’s request not to talk to a near-death patient about death” had the lowest (with a mean of 1.1 ± 82.37) severity of moral distress among nurses.

The mean score of ethical climate was 88.97, which indicated good ethical climate in the selected hospitals. Among the items related to the ethical climate questionnaire, the item of “I have an appropriate working relationship with my colleagues” had the highest score (with a mean of 4), and the item of “In this hospital, nurses are supported and respected” had the lowest score (with a mean of 2.65).

**Table 1:** Mean score of moral distress of nurses and their perception of ethical climate in terms of individual characteristics

**Individual ** **Characteristics**	**Frequency**		**Frequency of Moral ** **Distress**		**Severity of Moral ** **Distress**		**Statistical Test**
			**Mean**	***P*** **-Value**	**Mean**	***P*** **-Value**	
**Sex**						0.92	**Independent ** **T-Test**
Woman	220		41.1	0.002	50.3		
Man	80		47.8		50.1		
**Marital Status**						0.33	**Independent ** **T-Test**
Married	236		43.6	0.07	50.6		
Single	64		39.3		47.8		
**Employment Status**						0.34	**One-Way ** **Analysis-** **Variance**
Official	228		43.3	0.72	51.1		
Pseudo-Official	55		41.6		46.7		
Contractual	17		41.3		50.8		
**Ward**						0.027	**ANOVA**
Intensive	167		1.81	0.001	2.14		
Surgical	68		1.75		2.04		
Internal	20		2.27		2.44		
Pediatric	23		1.31		1.67		
Other	22		1.74		1.99		
**Passing Ethics Courses**						0.95	**Independent ** **T-Test**
Yes	155		1.89	0.004	2.09		
No	145		1.66		2.09		
**City**						0.46	**Independent ** **T-Test**
Tehran	205		42.6	0.63	50.8		
Isfahan	95		43.6		49		

The results of the present study showed no significant difference between nurses in Isfahan and Tehran regarding the frequency and severity of moral distress. Also, the total mean score of the frequency of moral distress was 1.0 ± 78.68, and the total mean score of the severity of moral distress was 2.09 ± 0.81. While the mean score of the frequency of moral distress was significantly higher among male nurses, the mean score of the severity of moral distress was not significantly different between male and female nurses. Also, no significant difference was observed between single and married nurses regarding the mean scores of frequency and severity of moral distress (Table 1).

There was no significant relationship between the means of frequency and severity of moral distress in nurses and their hospital of duty (*P* = 0.07), organizational position (*P* = 0.89), employment status (*P* = 0.44) and the type of overtime hours (*P* = 0.46). Also, nurses who had passed ethics courses had encountered morally distressful situations more often, but regarding the severity of moral distress, no significant difference was observed between nurses who had passed ethics courses and those who had not.

The results of the present study showed that the mean scores of frequency and severity of moral distress in nurses had a significant relation with their working department. In this regard, nurses working in the internal wards reported higher frequency and severity of moral distress (mean = 2.27) compared to nurses working in other wards. Also, pediatric nurses reported the lowest moral distress (mean = 1.31).

The results showed no significant relationship between the mean scores of frequency and severity of moral distress in nurses and their age, overtime hours, or work experience. Likewise, no relationship was found between the mean of the severity of moral distress and the nurses’ educational level and work experience. However, there was a direct relationship between the mean score of the frequency of moral distress and the nurses’ educational level, that is, with their educational level rising from bachelor’s to master’s degree and Ph.D., their perception of moral distress was improved (table 2).

**Table 2:** Pearson’s correlation coefficients between the severity and frequency of moral distress in nurses, and age, overtime, education level and work experience

**Score**	**Frequency of Moral Distress**		**Severity of Moral Distress**	
	***R***	***P***	***R***	***P***
Age	- 0.001	0.99	0.018	0.75
Overtime	0.027	0.63	- 0.007	0.90
Level of Education	0.218	< 0.001	0.009	0.87
Work Experience	0.016	0.78	0.018	0.75

According to the results, the mean score of ethical climate in the ISSO hospitals of Isfahan and Tehran was 88.97, which is at a good level based on the applied rating scale. As for the total mean score of ethical climate and its domains, there was no significant difference between the nurses of Isfahan and Tehran (*P* > 0.05). Also, in both cities, nurses had the most desirable viewpoint in the managers’ domain and the least desirable viewpoint in the patients’ domain (Table 3).

**Table 3:** Mean of total score of ethical climate and its domains by city of service

Score	Isfahan		Tehran		Independent T-Test	
	*Mean*	*Standard Deviation*	*Mean*	*Standard Deviation*	*T*	*P*
**Total Score of Ethical ** **Climate**	88.7	14.9	89.1	16.3	0.19	0.85
**Ethical ** **Climate/Colleagues**	15.4	2.6	15.3	2.6	0.30	0.77
**Ethical ** **Climate/Patients**	13.7	2.7	13.9	2.4	0.68	0.50
**Ethical ** **Climate/Managers**	21.3	5.2	22.2	5.5	1.24	0.21
**Ethical ** **Climate/Hospital**	19.7	3.8	19.1	4.2	1.08	0.28
**Ethical ** **Climate/Physicians**	18.6	4.3	18.6	5.2	0.03	0.98

The score of the frequency of moral distress in nurses had a reverse relationship with the total score of ethical climate, and the domains of colleagues, patients, hospital and physicians. However, no significant relationship was observed between the score of the frequency of moral distress and the score of ethical climate in the domain of managers. Also, the score of the severity of moral distress had a reverse relationship with the total score of ethical climate, and the domains of physicians and hospital (Table 4).

**Table 4:** Pearson’s correlation coefficients between severity and frequency of moral distress in nurses, and the total score of ethical climate

Score	Frequency of Moral Distress		Severity of Moral Distress	
	**r**	***P *** **value**	**r**	***P*** ** value**
**Total Score of Ethical ** **Climate**	- 0.194	0.001	- 0.170	0.003
**Ethical ** **Climate/Colleagues**	- 0.187	0.001	- 0.069	0.23
**Ethical ** **Climate/Patients**	- 0.181	0.002	- 0.053	0.36
**Ethical ** **Climate/Managers**	- 0.090	0.12	- 0.092	0.11
**Ethical ** **Climate/Hospital**	- 0.139	0.02	- 0.153	0.008
**Ethical ** **Climate/Physicians**	- 0.224	< 0.001	- 0.259	< 0.001

## Discussion

According to the results of the present study, the mean scores of frequency and severity of moral distress in nurses indicated moderate moral distress among participants from the selected ISSO hospitals. Also, the scores of moral distress in the nurses of Isfahan and Tehran were both at a moderate level, and no significant difference was observed between the nurses of these two cities.

The results showed that the severity and frequency of moral distress in nurses had a significant unilateral reverse relationship with the hospital ethical climate. This means that the more positive and favorable the ethical climate of a hospital is, the lower the severity and frequency of moral distress will be. Overall, the mean score of ethical climate in the selected hospitals of the ISSO in Isfahan and Tehran was at a good level. 

Different studies have reported different results regarding moral distress among nurses. Fernandez-Parsons et al. reported low moral distress among emergency nurses (25), while de Veer et al. found that nurses experience high levels of moral distress (5). A study by Abbaszadeh et al. reported a moderate level of moral distress and showed a significant relationship between the severity of moral distress and its recurrence (23).

Sile´n et al. demonstrated that although the severity of moral distress increased in situations where secure and appropriate care was not provided for the patient, the frequency and severity of nurses’ moral distress were still at a low level. In general, they reported that the frequency of moral distress was less than its severity. They also showed that a more positive perception of the ethical climate would decrease the frequency of morally distressful situations (12).

These differences in the results might be due to the different scales that were used in various studies on moral distress. Also, the items of the moral distress questionnaire may not sufficiently cover the moral concerns of the participating nurses, and even for those who have recognized their own distress, the items might cause too much discomfort, distress and disruption to answer realistically (6).

In studies that have reported higher levels of moral distress compared to the present study, there could be other personal and organizational factors causing moral distress to escalate among nurses; these factors may include the disproportion between the number of nursing personnel and the number of empty beds at the hospital, or the presence of unskilled physicians and managers.  Also in studies that reported lower perceived moral distress, these factors might have a certain quality that could consequently decrease the level of perceived moral distress among nurses (6).

In a study by Pinhero and De Souse in 2016 on operating room nurses with at least one year of work experience at the central hospital of Portugal, it was revealed that work environment and occupational satisfaction were at a desirable level and to improve and enhance this environment, the cooperation of the managerial team was necessary (26). Another study by Humphries and Woods in New Zealand showed that participants’ perceptions of the hospital ethical climate had been formed under the influence of interrelated factors (27). These factors included staffing levels, patient throughput (turnover) and the dynamics between the nursing staff and others within the workplace (27). Also in a 2007 study by Ulrich et al. conducted on 300 nurses across four different states of the United States, most of the participants evaluated the ethical climate of their workplace positive and higher than neutral with a mean score of 93 (2).

A 2016 study by Bartholdson et al. showed the nurses to have a weak perception of the ethical climate (28). This was due to inter-professional interactions, for instance the physicians’ attention to the opinions of nurses and assistant nurses, treatment-related decisions and respect for others’ opinions, especially at times of disagreement between different specialists on the best approach for the patient. After reviewing 32 articles, Schluter et al. revealed that weak ethical climate would escalate issues such as moral distress, insufficient or futile care, and unsuccessful or insufficient support for others, and might create false hope for the patients and their families (4).

According to the results, a significant relationship exists between the nursing service hospital and the ethical climate scores in the managers and hospital domains, but not the other domains. This indicates the effectiveness of the impact of management in the creation and development of a suitable and safe environment for the activities of the staff, which ultimately contributes to preventing or reducing moral distress among nurses and the adverse effects of these disturbances in the health care system.

The nurses who participated in this study expressed the most favorable opinion in the domain of managers, and the most unfavorable viewpoints in that of patients. In a study by Fazljoo et al. in 2014, the most favorable opinion pertained to managers and the most unfavorable viewpoint was related to physicians (29). Since nursing directors are chosen from among members of the nursing community and most of them have long-term clinical backgrounds, it seems reasonable that they should have a good understanding of the ethical atmosphere in this field. On the other hand, the hospitals affiliated with the ISSO are very crowded clinical centers in Iran, and providing health care for a large number of insured persons in this organization is very difficult and time consuming. Nurses are the most accessible responders to patients and are therefore required to address their needs and health expectations, which leads to inconveniences and problems in providing proper service and ultimately creates an undesirable view in this domain.

Of course, Health care organizations seem to be able to control the distresses by providing the nursing staff with ethical support and empowering them to offer quality care. It should also be noted that, managers and peers must also be willing to advocate for each other, should ethically difficult situations arise (4).

In the moral distress questionnaire, the highest frequency pertained to item 5 (Continue to participate in care for a hopelessly ill person who is being sustained on a ventilator, when no one will make a decision to withdraw support) with an average of 2.90. Conversely, item 2 (When the patient’s death is inevitable, I talk to the family about organ donation) had the lowest frequency with an average of 1.01. These findings are fully consistent with the results of other studies (10, 23, 30).

In general, review of the literature did not greatly change the results. The findings of different studies on nurses working in various clinical environments indicated that desirable ethical climate would decrease moral distress. A study by Pauly et al. in Columbia also showed that there was a significant relationship between nurses’ perceived moral distress and the hospital ethical climate, that is, improved ethical climate would decrease the perceived moral distress by nurses (6). Fry et al. also found that the more undesirable the hospital ethical climate was, the higher would the intensity of the perceived ethical distress and its complications in nurses be (14). Similarly, Fazljoo et al. showed a direct negative relationship between severity of perceived moral distress in nurses and ethical climate (29).

Based on the results of the present study, the mean scores of frequency and severity of moral distress was significantly lower among nurses who had selected nursing practice based on their own interest compared to those who had done so without interest. Borhani et al. also showed that the frequency and severity of moral distress were higher among nurses who had selected nursing practice without passion (8). According to their results, the mean scores of frequency and severity of moral distress were significantly lower among nurses who had never considered quitting their position or their profession compared to other nurses. These findings were in line with the results of other studies (2, 25, 31). A review study by Schluter et al. in 2008 demonstrated that moral distress and unfavorable ethical climate were causing a growing shortage of nursing personnel and an increase in nurses’ intention to transfer (4), and there is evidence indicating the effect of weak ethical climate on quitting the nursing practice. It seems that selecting nursing practice with passion could decrease nurses’ encounters with morally distressful situations due to better adjustment to the existing conditions (7). Although many researchers have stated that weak ethical climate and moral distress would cause nurses to leave the profession, the phenomenon has unfortunately not been accurately measured and reported. In fact, nurses’ decision to quit their profession due to moral distress and their perception of their workplace is still undetermined (4). If nursing practice is recognized as an ethical profession and nurses believe that they are performing an ethical act, the need for determining the effect of organizational barriers to performing the right action will be felt (32).

In the present study, all of the dimensions of ethical climate (colleagues, patients, hospital and physicians) had a significant relationship with moral distress, except for the dimension of managers. Also, the score of severity of moral distress had a significant reverse relationship with the total score of ethical climate and the dimensions of hospital and physicians. These results indicate that a series of factors are effective in nurses’ perception of ethical climate, and that a complex relationship exists between experiencing moral distress and the dimensions of ethical climate (6).

In the present study, the frequency and severity of moral distress varied in different wards. Thus, nurses working in the internal ward reported the highest level of moral distress compared to other nurses. The reason for this difference might be the fact that nurses of the internal ward would spend more time with their patients in comparison to nurses of other wards. Patients may be hospitalized in the internal ward for a long period of time, which can cause challenges to providing care. Also, the highest mean of severity of moral distress belonged to the item of “Due to the large number of patients, I cannot provide high quality care”. This indicates the occurrence of moral distress in situations where nurses are forced to provide care for a large number of patients in a short amount of time. A study by de Veer et al. in 2013 showed that nurses who face a shortage of time in providing care for their patients experience more moral distress, and the perceived pressure resulting from lack of time probably causes further concerns about the quality of the care they provide (5). Participants in the present study had the most desirable viewpoint in the managers’ dimension and the least desirable viewpoint in the patients’ dimension. Since nursing managers are part of the nursing community and mostly have a lot of experience working in clinics, it seems only natural that they should have a more desirable perception of the ethical climate in this dimension. On the other hand, the ISSO hospitals are some of the most crowded medical centers and providing service and care for such a large number of patients is exhausting if not intolerable. Nurses are required to fulfill all of the patients’ medical needs and expectations, and since they are the most available staff members, they are bound to encounter problems and experience dissatisfaction, and eventually develop undesirable viewpoints in this dimension. Organizations providing health services could control the dissatisfaction by providing guaranteed moral support for the nursing staff and empowering them to have control over the provision of quality care. Managers and other colleagues should also try to support each other in morally difficult situations (4).

The present study was conducted in the ISSO hospitals of Isfahan and Tehran. In Iran, the variables affecting the relationship between nurses’ moral distress and ethical climate have been examined by only one study conducted in hospitals covered by medical universities of Yazd. In the large cities of Iran, especially in Tehran, no similar study has been performed on the subject. The current study was done in the ISSO hospitals, which are rather different from university hospitals for reasons explained in the introduction section above. Therefore, to improve the generalizability of the results, it is recommended that future studies be conducted with larger sample size and participation in other large city hospitals.

## Conclusion

This study was an effort in the field of moral distress and ethical climate in the nursing community of the ISSO hospitals, and indicated that moral distress is closely related to hospital ethical climate. It is clear that determining strategies for decreasing the intensity and frequency of moral distress is an importance issue in these settings. Current study results showed the importance of creating a positive ethical climate to decrease moral distress in nurses and their tendency to leave their position or even the profession. This would lead to presentation of professional interventions for managing moral stress, increasing support for moral problems, creating appropriate communication within the organization and eventually decreasing moral distress in nurses. 

## Citation to this article:

Bayat M, Shahriari M, Keshvari M. The relationship between moral distress in nurses and ethical climate in selected hospitals of the Iranian social security organization. J Med Ethics Hist. 2019; 12: 8.
